# Type 2 Diabetes Contributes to Altered Adaptive Immune Responses and Vascular Inflammation in Patients With SARS-CoV-2 Infection

**DOI:** 10.3389/fimmu.2022.833355

**Published:** 2022-03-24

**Authors:** Manpreet Singh, Obed Barrera Adame, Michael Nickas, Jeremiah Robison, Christopher Khatchadourian, Vishwanath Venketaraman

**Affiliations:** ^1^St. Barnabas Hospital Health System, Department of Emergency Medicine, Bronx, NY, United States; ^2^Western University of Health Sciences College of Osteopathic Medicine of the Pacific-Pomona, Pomona, CA, United States

**Keywords:** type 2 diabetes, SARS-COV-2 infection, immune response, inflammation, oxidative stress

## Abstract

SARS-CoV-2, which initially emerged in November of 2019, wreaked havoc across the globe by leading to clinical acute respiratory distress syndrome and continues to evade current therapies today due to mutating strains. Diabetes mellitus is considered an important risk factor for progression to severe COVID disease and death, therefore additional research is warranted in this group. Individuals with diabetes at baseline have an underlying inflammatory state with elevated levels of pro-inflammatory cytokines and lower levels of anti-inflammatory cytokines, both of which cause these individuals to have higher susceptibility to SARS- CoV2 infection. The detrimental effects of SARS-CoV-2 has been attributed to its ability to induce a vast cell mediated immune response leading to a surge in the levels of pro-inflammatory cytokines. This paper will be exploring the underlying mechanisms and pathophysiology in individuals with diabetes and insulin resistance making them more prone to have worse outcomes after SARS- CoV2 infection, and to propose an adjunctive therapy to help combat the cytokine surge seen in COVID-19. It will also look at the immunomodulatory effects of glutathione, an antioxidant shown to reduce immune dysregulation in other diseases; Vitamin D, which has been shown to prevent COVID-19 patients from requiring more intensive care time possibly due to its ability to decrease the expression of certain pro-inflammatory cytokines; and steroids, which have been used as immune modulators despite their ability to exacerbate hyperglycemia.

## Introduction

With approximately 251 million confirmed cases, over 5 million deaths documented and only about 3.15 billion of the 7.7 billion total global population fully vaccinated as of November, 2021 (1). The novel strain of coronavirus, which originally appeared in Wuhan, in the People’s Republic of China at the end of 2019 and was found to precipitate acute respiratory distress syndrome (ARDS) is still wreaking havoc across the globe (2).

The genetic similarity of this novel strain to SARS-CoV-1, formerly known as SARS-CoV, and Middle East Respiratory Syndrome Coronavirus (MERS-CoV) lead to it being named Severe Acute Respiratory Syndrome Coronavirus 2 (SARS-CoV-2). SARS-CoV-2 is an enveloped, positive-stranded RNA virus which belongs to a large family of Coronaviridae viruses with noxious capability across many species (3). Prior to COVID-19, Coronaviridae family was well known for being the most common cause of common cold.

Evolution and mutations are an essential part of the viral life cycle of SARS-CoV-2; therefore, the risk of a more virulent strain or decreased vaccine efficacy is a potential outcome. Since the emergence of SARS-CoV-2 there have been a few different variant strains, some more prevalent in certain regions. The World Health Organization (WHO) has separated these variants into four different classes based on their potential for harm, those deemed to be a lower risk were assigned to the class: variant being monitored (VBM), next is variant of interest (VOI), followed by variant of concern (VOC) and finally variant of high consequences (VOHC). The designation of a variant to a certain class differs from country to county (4). Currently, WHO has designated five of the SARS-CoV-2 variants as VOC, these include Alpha, Beta, Gamma, Delta and the newest variant Omicron (5). For a variant to be assigned to VOC this conveys that this strain has shown to be highly transmissible, has increased morbidity and mortality, has shown to have a reduction in neutralization by antibodies, has decreased vaccine or treatment effectiveness as well as interference with diagnostic testing (6). The precise impression is yet to be determined for variant Omicron as this strain materialized very recently and research is ongoing. All of the VOCs have mutations in the spike protein, with variant Alpha having mutations in receptor binding domain along with spontaneous spike mutation and mutation near S1/S2 furin cleavage site, Beta variant was found to have mutations in receptor binding domain of spike protein and Gamma and Delta variants both demonstrated numerous spike protein mutations. As more information becomes available, Omicron was found to have spike protein mutations- a protein essential for viral entry and hence has been a target for vaccine development (7–9).

**Figure 1:**
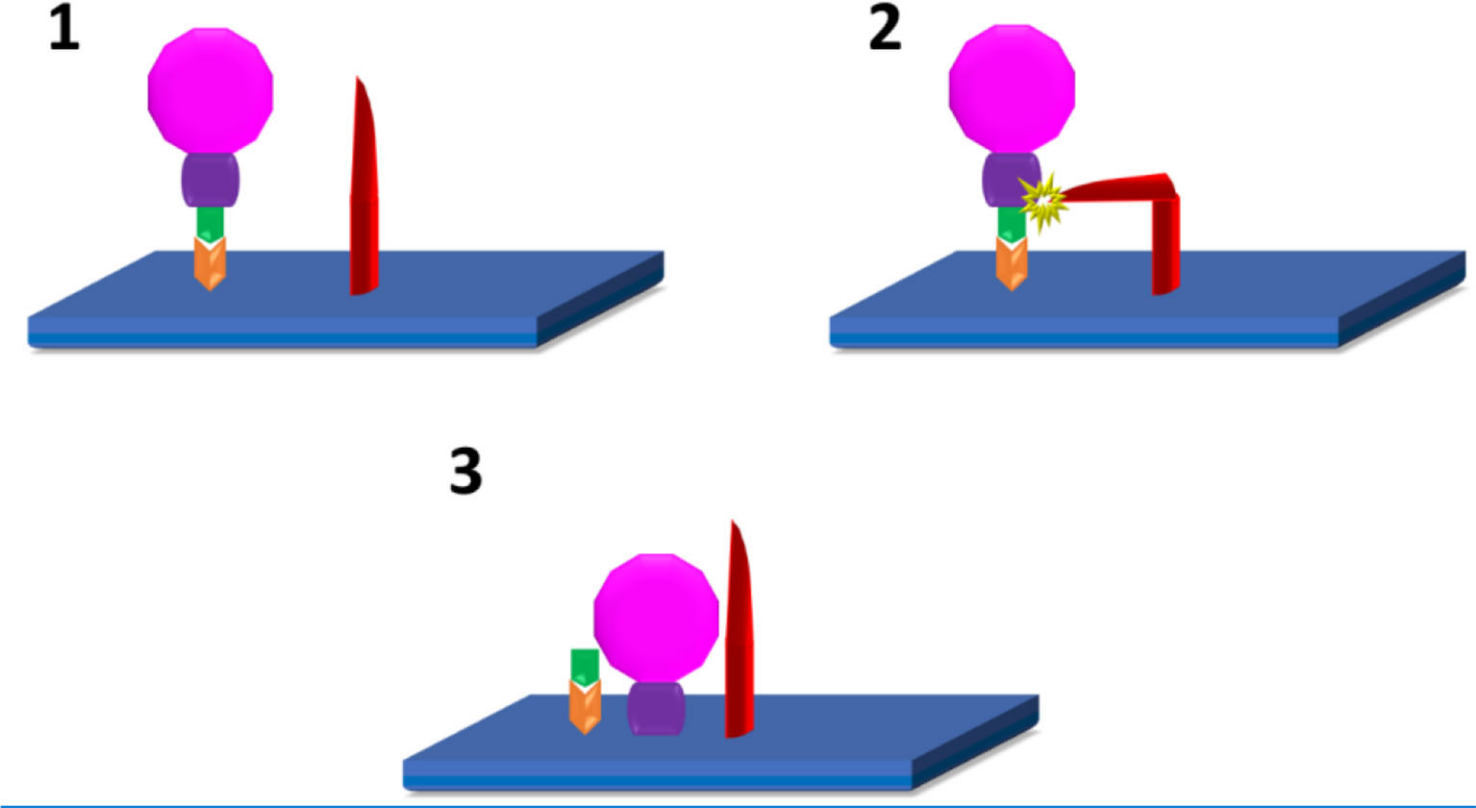
SARS-CoV2 infection of host cells (10–12): 1. Attachment of S1(green) subunit of the SARS-CoV-2 (pink) spike protein to the ACE 2 (Orange) receptor on the host cell plasma membrane (blue). 2. Cleavage of S1/S2 (green/purple) complex by either TMPRSS2, or Furin (red) 3. Fusion of the S2 spike protein component with the host cell plasma membrane.

Over the past year there have been many different mechanisms of virulence, pathophysiology and mechanism of action proposed, each changing as newer information has become available about this novel strain. The mode of infection is *via* the viral spike S-glycoprotein binding to the ACE2 (**Figure 1**) on the epithelial surface to mediate viral entry (13). This process is supported by transmembrane protease serine 2 (TMPRSS2) (14), which in COVID-19 leads to a robust immune response resulting in acute inflammation with increased levels of inflammatory mediators including cytokines and chemokines (15). This activation of the immune system provides us with the insight as to why individuals who are immunocompromised at baseline with other comorbidities such as diabetes tend to have a more severe disease progression. Multiple studies have shown that individuals with diabetes are prone to have cytokine dysregulation at baseline with increased levels of pro-inflammatory cytokines and decreased levels of anti-inflammatory cytokines (16).

The difficulties attributed to COVID-19 response is multi-factorial, some of which include new emerging strains, lower socioeconomic status, lack of resources and different co-morbidities with underlying augmented baseline inflammation and immune dysregulation as seen in diabetes. This paper will be divulging into the mechanism and pathophysiology of why individuals with diabetes and insulin resistance are more prone to have worse outcomes after COVID-19 and propose an adjunctive therapy to help combat the cytokine storm as seen in COVID-19 to help alleviate the underlying inflammation (2, 16).

## Pathogenesis of SARS-CoV-2

SARS-CoV-2 are RNA viruses which spread *via* bodily fluid exposure. Viral attachment and entry into host cells happens using the spike protein which sits on the virus’ membrane. Spike protein is composed of two subunits S1 which promotes infectivity *via* receptor binding and S2 which increase entry *via* endocytosis. S1 subunit which infects *via* receptor binding attaches to the host cell *via* angiotensin-converting enzyme-2 (ACE2) receptors (**Figure 1**). Here the entire virus enters the cell by being encased in an endosome. Once in the cell, this membrane is then broken-down piece-by-piece by the protease cathepsin. Eventually, this will expose the viral RNA to the cytoplasm. Neuropilin-1 is another host cell receptor for SARS-CoV-2 and facilitates infectivity, the exact mechanism is still debatable, though it is thought to allow for S1 binding as well (16–22).

The second mode of entry requires spike protein cleavage at the S1/S2 site by proteases which includes transmembrane protease serine 2 (TMPRSS2) and cathepsin L (16, 17). This allows for fusion of the S2 component with the cell’s plasma membrane leading to an opening or fusion pore, facilitating the RNA’s entry into cellular cytoplasm (17, 21, 22). Once in the cytoplasm of the cell the RNA genome gets translated into viral proteins. These will eventually form into viruses and be released from the cell (23). **Figure 1** demonstrates the steps for entry requiring spike protein cleavage.

As more information about this novel virus is elucidated, another protease’s role in promoting infectivity is becoming more significant. Furin is a proprotein convertase (PC) that has a role in cleaving proteins. It is generally located on the trans-Golgi network and has been found to also cleave at the S1/S2 component of the spike protein. It is thought that this will enhance viral maturity and processing once the virus is already in the cell (23, 24). The spike protein also has a unique furin cleavage site at the S1/S2 junction which facilitates fusion of S2 to the cellular receptor neuropilin-1 (NRP1). This amplifies transmission of SARS-CoV-2 (25). Studies show that patients with diabetes and metabolic syndromes have higher plasma furin levels (26).

It has further been discussed that this elevation in furin may increase the mortality of COVID-19 patients. The augmented furin levels raises susceptibility of host cells, hence boosting viral load. We predict that patients may have worse outcomes secondary to higher viral infiltration of organs with higher furin concentration such as the lungs, kidneys, and atherosclerotic plaques. Respectively, many fatal outcomes from COVID-19 seem to come from acute respiratory distress syndrome, acute renal failure, and acute cardiac injury with heart failure (27).

## Mechanism for Insulin Resistance

Diabetes mellitus is a disorder affecting glucose metabolism and can be separated into two types. Type one involves insulin deficiency while the more common type two involves insulin resistance (28, 29).

Insulin resistance is a decreased responsiveness of tissues and cells to circulating insulin. This leads to both increased circulating levels of insulin, as well as higher levels of circulating glucose (30).

Insulin resistance is a metabolic disorder that is not easily explained by a single metabolic pathway. Chronic exposure to energy surplus is postulated to lead to insulin resistance. This usually induces ectopic lipid accumulation in the liver, muscles and adipose tissues leading to reduced glucose uptake by skeletal muscle and decreased glycogen synthesis in the liver (28). Ectopic lipid accumulation is postulated to be one of the main causes of development of insulin resistance. Early studies by Randle suggest that fatty acids impair insulin-mediated glucose uptake by inhibition of pyruvate dehydrogenase, leading to decreased glucose oxidation, which is necessary for glucose metabolism (29).

While plasma lipids play an important role in insulin resistance, other studies have demonstrated that elevated accumulation of intra-myocellular lipid, especially diacylglycerol (DAG) alters the cellular matrix by activating a *de-novo* protein kinase C (PKC). This impairs insulin signaling by increasing protein phosphatase 2A (PP2A) and sequestrating serine/threonine kinase (Akt2); both of which play an important role in the function of the insulin receptor and it’s signaling pathway as well as in glucose uptake (31–33).

Ceramides are by-products of fatty acids and are believed to play an important role in insulin resistance. As fatty acids enter the cell, they get esterified with sphingosine creating ceramides. These ceramides are membrane bound lipids, found in greater quantities in the muscles and liver cells (34). One-way chronic inflammation can be explained in patients suffering from insulin resistance is by the activation of the innate immune signaling pathway triggered by toll-like receptor 4 (TLR4). TLR4 are an upstream signal required for ceramide biosynthesis. Ceramides are associated with lipid-induced inflammatory pathways and the development of proinflammatory components such as kinase IKβ (35). Patients in septic shock who are suffering from obesity and/or diabetes exhibited an increase in insulin resistance and an elevation in cytokines, this would suggest that insulin resistance plays a role in cytokine production and its downstream complications (36, 37). In turn, cytokines can feed back into this system causing insulin resistance. For example,Tumor necrosis alpha (TNF-α) directly causes insulin resistance through insulin receptor substrate-1 (IRS-1) and insulin receptor substrate-2 (IRS-2 *via* promoting serine/threonine phosphorylation of the substrates. This leads to an inhibition of the insulin receptor and causes insulin resistance (38).

Diabetes also imparts damage to the body by increasing oxidative stress in DNA, proteins and lipids, which leads to an enhanced cellular response and activation of PKC, transcription factor nuclear factor kappa B (NF-κB) and JNK stress associated kinase (39). Hyperglycemia also leads to microvascular damage, resembling poorly perfused tissue. This in part is referred to as pseudohypoxia which impairs oxidation of NADH to NAD+. Oxidative stress increases reactive oxygen species (ROS) such as superoxide anion and possibly nitric oxide formation and decreases oxidative defenses leading to tissue damage (40).

While there are multiple pathways which lead to oxidative stress such as glucose oxidation, non-enzymatic glycation, and polyol pathways. The polyol pathway appears to have the greatest effect on oxidative stress in an individual (41). The polyol pathway consists of two enzymes, aldose reductase (AR) and sorbitol dehydrogenase (SDH), that aid in the formation of glucose to sorbitol and sorbitol to fructose (42). Therefore, hyperglycemia leads to tissue damage *via* two pathways, osmotic damage *via* sorbitol accumulation and oxidative stress by means of oversaturating the polyol pathway and ROS accumulation. The first reaction requires a NADPH for the conversion of glucose into fructose by AR. This in turn, depletes NADPH which is needed by glutathione reductase to generate glutathione (GSH), leading to a depletion of GSH. The second reaction is the conversion of sorbitol to fructose by SDH which causes oxidative stress by increasing the NADH cofactors, a substrate for ROS (43). These are some of the main pathways and reactions believed to cause tissue damage, increase pro inflammatory molecules which eventually leads to insulin resistance.

## Mechanism Behind Impaired Immune Response in Diabetes

The pathophysiology and treatment of SARS-CoV-2 has been inexorably linked with our immune system. Severe SARS-CoV-2 infections appear to be largely related to inappropriate and excessive immune system response (15). Meanwhile, humoral and cell mediated immune responses appear to be the cornerstone of the treatment and prevention of SARS-CoV-2. Many attempts at therapeutics have relied on convalescent plasma or monoclonal antibodies, supporting, and amplifying our body’s own natural immune system. Likely more important, highly effective COVID-19 vaccines rely on our bodies own humoral immune system to be effective.

Diabetic patients, making up over ten percent of the population in the United States, and known to have markedly increased mortality rates due to SAR-CoV-2 infections are a group of significant interest regarding COVID-19 infections (4, 44). A meta-analysis concluded that blood glucose level was elevated in severe SARS-CoV-2 when compared to milder disease, and while hemoglobin A1C (HbA1C) was slightly higher in severe disease, it was not significant. This was again seen in a retrospective study by Patel and team which showed that in diabetic patients hospitalized with SARS-CoV-2 there was no significant association between HbA1C level and adverse clinical outcomes (45, 46). Despite there being no significant correlation between HbA1C and adverse outcomes early in the pandemic, a recent meta-analysis by Zheng et al. concluded that uncontrolled hyperglycemia for a prolonged period was associated with adverse prognosis. The mechanistic effect of hyperglycemia on inflammation and cytokine surge has been established in many studies (47).

Glucose reacts non-enzymatically with proteins, forming compounds which undergo a series of irreversible changes over time to become advanced glycation end-products (AGEs). In studies AGEs significantly increase the mRNA expression and production of TNF- α in endothelial cells cultured and grown from the human umbilical vein. TNF- α can then induce the production of inflammatory cytokines such as interleukin-6 (IL-6), interleukin-8 (IL-8), and granulocyte-macrophage colony-stimulating factor (GM-CSF) from endothelial cells (48).

Diabetes, poor glycemic control, and inability to produce adequate insulin are related to several immunosuppressive effects in relation to B and T cell activity, and lead to the disease being considered an immunosuppressive condition. These effects are likely the root of the markedly increased mortality seen in this population, and worth further investigating (44).

Cell-mediated response is assisted and amplified by the activity of CD8+ T cells, leading to the cascade of cytokines that rally other components of the cell-mediated immune response. Prior research has shown that this response is blunted in diabetics (46, 49). For example, research has shown that viral infection can cause states of insulin resistance in skeletal muscle and other sites in the body. This leads to a hyperglycemia and resulting compensatory hyperinsulinemic state. These increased levels of insulin have been shown to be stimulating factors for CD8+ T cells. This hyperinsulinemic response has been shown to be blunted in patients with Type II diabetes (T2DM), leading to decreased CD8+ T cell activity and a state of hyperglycemia in the presence of infection (50, 51). There are also associated effects of uncontrolled hyperglycemia, including glycosylation components of the immune system including IgG and IgM antibodies, as well as inappropriate activation of CD4+, CD8+, and cytotoxic T cells, and decreased production of inflammatory cytokines in immune responses (52). A study comparing patients with impaired glucose tolerance (IGT) to non-diabetic subjects showed that IL-6, C-reactive protein (CRP), serum amyloid A protein, and fibrinogen were more elevated in patients with T2DM (53). Another study looked at type II diabetics who did and did not have an infection present compared to non-diabetic patients with and without infection. Both cohorts of diabetics had higher levels of CRP than the control group of non-diabetic patients with no infection (54, 55).

SARS-CoV-2 infections present somewhat of a paradox regarding cell-mediated immune response. While necessary to control the viral replication and limit infection, it appears most of the serious adverse effects of the infection result from an overactive cytokine cascade. This should raise the question of why immunosuppression is not beneficial in these patients. A similar issue exists regarding steroid use as a therapeutic agent, having been found to have both beneficial and harmful effects in different patient populations (56). Likely the easiest way to parse out the complicated relationships between these competing effects is to look at the result. Diabetics are found to have a higher rate of clinically significant infection, and nearly 3 times the mortality rate from infections (44). This suggests that while there are likely both protective and deleterious effects of the blunted immune response in diabetics- overall it is a detrimental process. The differential effects of glucose on the various immune cell types can be gleaned in **Table 1**.

**Table 1:** The effects of hyperglycemia on the various types of immune cells.

Immune Cell Type	Effects of Glucose on the Cell Type
Monocytes	Il-6 and TNF- α levels are increased in human-isolated monocytes (57). Superoxide anion production is increased (58)
Neutrophils	Inhibits migration (58, 59), phagocytosis, superoxide production and microbial killing, and induces apoptosis (58)
Lymphocytes (B cells)	Glycosylation of proteins prevents complement fixation and subsequent opsonization with immunoglobulins (58)
Macrophages	Superoxide anion production is increased (59)
Lymphocytes (T cells)	Attenuated CD28 and CD3 signaling leads to diminished response in activated T cells. Activity and counts of regulatory T cells are reduced (59)
Natural Killer Cells	Attenuated function and cell count (59)

Humoral immunity, and a sustained antibody response to SARS-CoV-2 infections, whether by natural infection or vaccination appear to be the definitive method of control of the pandemic. The question of sustained immune response will likely be the deciding factor of whether COVID-19 becomes a seasonal infection, or eventually sputters out as the number of potential infected dwindles. One of the current areas of interest is how long the antibody response to COVID-19 infections lasts from a natural infection, how long it provides effective resistance, and how the response from different vaccines compares to this (60). Diabetics are again a group of interest because of impaired B-cell response. Clinically, diabetics have shown impaired humoral immune responses to both infections and vaccinations in the past (significant examples being influenza and hepatitis B vaccines) (61). The effects of diabetes on humoral immunity are complex and interconnected. Factors limiting cell mediated immune responses mentioned above contribute to a less robust activation of the humoral immune system, while glycosylation of antibodies directly affects the effectiveness of these responses (52, 61). All of this inflammatory dysregulation present in diabetics is a recipe for disaster if mixed with COVID-19. Both disease states have many similar pro-inflammatory mechanisms that can potentiate a very sick patient (50). Despite this, clinical trials have shown mixed, but promising, results of immune response to COVID-19 (measured in antibody titers) that appear to be appropriate and sustained (61, 62). Giving some hope that this population will have an effective response to vaccination programs going forward. **Table 2** Summarizes the main factors of diabetes that contribute to SARS-CoV-2 pathophysiology as discussed in this paper.

**Table 2:** Summary of the main factors of diabetes that contribute to SARS-CoV-2 pathophysiology as discussed in this paper.

Summary of hyperglycemic factors that contribute to SARS-CoV-2 pathophysiology	Mechanism
CD8+ T Cells	• Role in enhancing the cytokine cascade (49)
TNF- α	• Induces production of inflammatory cytokine
	• Causes insulin resistance by inhibition of insulin receptor, hence leading to hyperglycemia and worsening inflammation (34, 49)
Furin	• Noted to be elevated in diabetic patients, plays a role in host cell susceptibility to SARS-CoV-2 (26, 27)
Inflammatory Cytokine: IL-6	• Elevated in diabetic individuals, role in augmentation of COVID-19 inflammatory state (54, 55).

## Role of Vitamin D and Glutathione in Decreasing Cytokine Surge and Oxidative Stress

Glutathione (GSH) is an endogenous compound which continuously cycles between oxidative states in order to regulate the body’s oxidation status (63). Reduced GSH is oxidized to form Glutathione disulfide (GSSG) upon reduction of reactive oxygen species (ROS)-scavenger enzymes (64). Thus, GSH ultimately neutralizes oxidative compounds *via* a series of enzymatic reactions, combating the oxidative stress induced pro-inflammatory cytokine expression classically seen in diabetic patients (63, 64). Certain agents can upregulate or downregulate glutathione activity. For instance, N-acetyl-cysteine (NAC) and glutathione ether ester (GEE) increase intracellular GSH levels whereas Diamide and Buthionine Sulfoxime (BSO) deplete intracellular GSH (64–66). In lymphoma B cells, BSO treatment and consequent GSH depletion increased ROS production, ultimately inducing apoptosis (65).

GSH affects the expression of cytokines, this has been established by many studies which show an imbalance between pro and anti-inflammatory cytokines in disease states such as diabetes and human immunodeficiency virus (67). In a study analyzing heart failure among rabbits, those treated with NAC showed greater levels of GSH and consequently decreased expression of the transcription factor NF-κB when compared to controls. NF-κB activity upregulates expression of IL-6, interleukin-1(IL-1), and TNF-α (66). Furthermore, lower total GSH levels were correlated with increased disease severity and lung damage, highlighting glutathione’s potential role in COVID-19 management (68). Essentially, GSH reduces oxidative stress and the expression of cytokines pertinent to the diabetes related exacerbation of SARS-CoV-2 pathogenesis and thus could be a potential therapeutic target for reducing disease severity. Several clinical trials, as listed in **Table 3**, investigate the therapeutic potential of glutathione in COVID-19 pathogenesis; however, diabetic-specific trials have not yet been commenced (69–73).

**Table 3:** Current glutathione-relevant clinical trials in COVID-19 patients and their corresponding clinicaltrials.gov identification numbers, treatments, measured parameters, and clinical phases.

Trial Title	Identification Number	Treatment	Measurements	Clinical Phase
Glutathione, Oxidative Stress and Mitochondrial Function in COVID-19	NCT04703036	N-Acetylcysteine	Various cytokines including IL-6, oxidative stress, patient functional status, and markers of damage	
A Study to Evaluate the Efficacy and Safety of Prothione™ Capsules for Mild to Moderate Coronavirus Disease 2019 (COVID-19)	NCT04742725	Prothione™ (a pro-glutathione compound)	time from RT-PCR SARS-CoV-2 positivity to double-RT-PCR negativity	Phase 2
Effect of N-acetylcysteine on Oxidative Stress in COVID-19 Patients	NCT04792021	N-Acetylcysteine	IL-6, TNF-α, Length of stay (LOS) in hospital, and mechanical ventilation requirement	Phase 3
NAC for Attenuation of COVID-19 Symptomatology (NACinCOVID2)	NCT05074121	N-Acetylcysteine	COVID disease symptom severity and duration	Phase 2
Efficacy of N-Acetylcysteine (NAC) in Preventing COVID-19 From Progressing to Severe Disease	NCT04419025	N-Acetylcysteine	LOS in hospital, respiratory rate, mechanical ventilation requirement, required duration of intubation, COVID-19 hospitalization	Phase 2

In addition to GSH, Vitamin D can also be implicated in the therapeutic barrage against COVID-19. In a randomized clinical study, patients with COVID-19 who were administered 25-Hydroxyvitamin-D3 (calcifediol) showed a significantly lower need for Intensive care unit (ICU) treatment when compared to non-administered COVID-19 patients (74). In another randomized clinical trial consisting of 130 COVID-19 patients with hypovitaminosis D, high dose (60,000 IU) daily oral supplementation of Vitamin D significantly increased systemic Vitamin D levels and decreased the levels of the following inflammatory markers: C-reactive peptide (CRP), IL-6, and Neutrophil/lymphocyte ratio (75). Information on clinical trials involving Vitamin D supplementation in COVID-19 patients can be gleaned from **Table 4** (76, 77). No clinical trials involving vitamin D supplementation in diabetic COVID-19 patients have been undertaken.

**Table 4:** Current Vitamin D-relevant clinical trials in COVID-19 patients and their corresponding clinicaltrials.gov identification numbers, treatments, measured parameters, and clinical phases.

Trial Title	Identification Number	Treatment	Measurements	Clinical Phase
The Effect of Vitamin D Therapy on Morbidity and Mortality in Patients With SARS-CoV 2 Infection	NCT04733625	Cholecalciferol	Death and need for intubation	
Clinical Outcomes of High Dose Vitamin D Versus Standard Dose in COVID-19 Egyptian Patients	NCT04738760	High dose (dose not provided) Vitamin D	LOS in hospital, mortality, Clinical status improvements, the rate and magnitude of change in gas exchange as measured by PaO2/FiO2 ratio	Not Applicable

Both 25-Hydroxyvitamin-D3 and 1,25-Dihydroxyvitamin-D3 (calcitriol) inhibit the expression of TNF-α, IL-6, Monocyte chemoattractant protein-1(MCP-1) and NF-κB in lipopolysaccharide-stimulated and unstimulated macrophages (78). They also significantly reduce the expression of IL-6, MCP-1, and IL-8 in a dose-dependent manner among human periodontal ligament fibroblasts as well as the expression of MCP-1 and IL-8 in primary human periodontal ligament cells (79). These anti-inflammatory outcomes of vitamin D administration may be explained by its effects on TNF-α. In vascular smooth muscle cells (VSMCs) TNF-α activates NF-κB *via* TNF receptor binding, eventually inducing the expression of pro-inflammatory genes such as MCP-1 and Interleukin -1 beta (IL-1β) (80). However, administration of both calcitriol and paricalcitol attenuated TNF-α-dependent NF-κB binding to DNA, decreasing nuclear translocation of NF-κB and consequent NF-κB-induced gene transcription (81). Thus, vitamin D inhibits the NF-κB-induced transcription of cytokines upregulated during hyperglycemia and the cytokine storm seen in severe SARS-CoV-2 infection. Vitamin D may also alleviate heightened oxidative stress characteristic of hyperglycemia, subsequently alleviating SARS-CoV-2’s pathogenesis in diabetic patients. According to a meta-analysis and systematic review by Sepidarkish et al., Vitamin D supplementation increases serum levels of glutathione (GSH) and significantly depletes malondialdehyde (MDA), indicating decreased oxidative stress (82). Furthermore, treatment with Vitamin D increases glucose tolerance and decreases insulin resistance. The proposed mechanism involves a direct stimulation of insulin-producing pancreatic cells *via* their vitamin D receptors and an indirect stimulation *via* normalization of systemic calcium levels, allowing appropriate calcium-induced insulin secretion (83). Despite there being no clinical trials demonstrating that low Vitamin D levels lead to higher SARS-CoV-2 susceptibility, there have been numerous studies showing that the pre-infection Vitamin D deficiency is associated with increased morbidity and mortality and hence lower Vitamin D levels are likely a good predictor for disease progression (84).

A study by Demir et al. validates the impact of Vitamin D on inflammation. This study showed that as Vitamin D levels increased, the risk of SARS-CoV-2 infections decreased along with its downstream effects. It proved that if an individual is already positive for COVID-19, as their Vitamin D levels increased, the levels of inflammatory markers such as D-Dimer and C-reactive protein decreased along with the extent of lung injury (85).

Numerous studies have displayed that having baseline levels of Vitamin D deficiency is a common risk factor for multiple inflammatory diseases including obesity, diabetes, insulin resistance and atherosclerosis (86, 87).

A study conducted by Jain and team looked at the link between Vitamin D deficiency and a lower GSH level, where they showed that supplementation using a combination of Vitamin D and L-cysteine significantly raised GSH levels while lowering oxidative stress, TNF-α level, and insulin resistance levels in blood (86).

Thus, Vitamin D supplementation not only reduces oxidative stress, but it also augments GSH and contributes to improving diabetic hyperglycemia and its associated inflammation.

## Steroid’s Role in COVID-19 Infection

Corticosteroids which include glucocorticoids and mineralocorticoids are a class of stress hormones released in a circadian manner by the adrenal cortex. It plays a role in regulating physiologic processes essential for life (88). Today corticosteroids are at the forefront of immunosuppressive and anti-inflammatory therapy and hence are a critical part in the fight to combat the detrimental impact of SARS-CoV-2 (89). The release of glucocorticoids is regulated by hypothalamic-pituitary-adrenal (HPA) axis (88, 90). The downstream effects of glucocorticoids are mediated *via* two major pathways; first by its direct effect on gene transcription *via* glucocorticoid responsive elements and transcription factors, both of which inhibit cytokine synthesis, obstruct promoter sites of pro-inflammatory cytokines and impede nuclear factor kappa B. Nuclear factor kappa B which acts to activate immunoregulatory genes in inflammation, therefore when inhibited by steroids, leading to a decrease in inflammation (91, 92). Secondly corticosteroids modulate cytokine function *via* post translation events by affecting the stability of messenger RNAs which encodes for certain cytokines (93, 94).

Corticosteroids are immunosuppressants and hence will function to decrease the cytokine production, but concurrently would also hinder the defensive properties of the immune system by blocking T-cells and decreasing the ability of B-cells to make antibodies. They also subdue the function of macrophages and natural killer cells, hence, impeding the body from fighting against superimposed infections (95). A study by Lee et al. showed that early corticosteroid use was associated with higher continuous plasma viral load (96). The recovery trial, one of the more prominent studies supporting corticosteroids for SARS-CoV-2 concluded that dexamethasone for 10 days reduced 28-day mortality in patients receiving some form of respiratory support but found no benefits and possibly harm in patients not requiring oxygen supplementation. This was also seen documented in the meta-analysis by WHO prior to their recommendation for corticosteroid use along with many other studies, all of which recommended corticosteroids for severely ill patients only (10, 56, 95, 97, 98).

As SARS-CoV-2 lead to devastation across the globe, the impact of different co-morbidities became more apparent, and the different consequences became evident. Diabetes is one of the co-morbidities that has been shown to be an independent risk factor for infections and for severe disease progression of Covid-19 (11, 12). There have been many emerging reports and studies showing that there is a high rate of mucormycosis infection in patients with SARS-CoV-2 in India. Hyperglycemia, becoming worse in the setting of corticosteroid supplementation, provides an ideal environment for fungal infections (11). In a systemic review of cases looking at 101 patients with mucormycosis it was found that 80% were diabetic patients and 76.3% had received corticosteroids, as there was no specific documentation of the prevalence of infections in diabetic and non-diabetic patients’ definitive conclusions could not be drawn (11).

As noted above, steroids are undoubtfully beneficial and most definitely contributed to keeping our mortality rate lower, but are not without risk, and these risks are compounded when coupled with certain co-morbidities such as diabetes. Therefore, it is crucial that the usage of corticosteroids is done cautiously in certain population, such as the immunocompromised community.

## Conclusion

Diabetes is currently one of the top ten causes of death worldwide, the number of individuals with diabetes rose from 108 million in 1980 to 422 million in 2014 and in 2019, an estimated 1.5 million deaths were directly caused by diabetes (1). The underlying immune system dysregulation became an imminent point of interest as the COVID-19 pandemic swept through the globe. SARS-CoV-2 by itself had exhausted medical resources as there was a rush to analyze the pathogenesis of the novel virus. Nowadays, there are multiple vaccines available but only a fraction of the world’s population has been vaccinated. As we see newer strains emerging it begs the question if the current vaccines provide appropriate protection from these evolving viruses.

In the executive summary published by the Centers for Disease Control and Prevention (CDC), reviewing the meta-analysis of clinical trials, it was suggested that vaccination is effective in preventing severe infection and provided protection from variants. This analysis also showed that various immune markers including neutralizing antibodies, including CD 4+ and CD8+ T cells, have a half-life, and see a decline after a period of time. Memory B cells on the other hand was elevated after an infection/vaccination and this level was sustained (99, 100).

CDC’s weekly report showed that vaccine effectiveness was significantly reduced in patients 4-6 months after vaccination, showing increased morbidity and mortality among nursing home residents who had already received the full vaccination series when the Delta variant predominated, hence reenforcing the need for boosters (101).

Here we presented GSH, an antioxidant that has been shown to normalize pro and anti-inflammatory cytokines in other disease states such as diabetes and HIV, as a potential therapeutic pathway for COVID-19 infections. We also discussed the risks and benefits of corticosteroids and recommend that physicians use them sparingly when dealing with patients who do not require respiratory support. Immunomodulation with steroids, GSH, and Vitamin D have shown beneficial effects in prior studies related to similar inflammatory conditions and warrant further research in the pathophysiology and treatment of COVID-19.

## Author Contributions

VV and MS conceived this work. All the authors contributed to draft of the manuscript. OB, MN, and JR contributed equally to this manuscript. All authors contributed to the article and approved the submitted version.

## Conflict of Interest

The authors declare that the research was conducted in the absence of any commercial or financial relationships that could be construed as a potential conflict of interest.

## Publisher’s Note

All claims expressed in this article are solely those of the authors and do not necessarily represent those of their affiliated organizations, or those of the publisher, the editors and the reviewers. Any product that may be evaluated in this article, or claim that may be made by its manufacturer, is not guaranteed or endorsed by the publisher.
